# Quantitative Cartilage Imaging in Knee Osteoarthritis

**DOI:** 10.1155/2011/475684

**Published:** 2010-12-08

**Authors:** Felix Eckstein, Wolfgang Wirth

**Affiliations:** ^1^Institute of Anatomy and Musculoskeletal Research, Paracelsus Medical University, Struberga*β*e 21, 5020 Salzburg, Austria; ^2^Chondrometrics GmbH, 83404 Ainring, Germany

## Abstract

Quantitative measures of cartilage morphology (i.e., thickness) represent potentially powerful surrogate endpoints in osteoarthritis (OA). These can be used to identify risk factors of structural disease progression and can facilitate the clinical efficacy testing of structure modifying drugs in OA. This paper focuses on quantitative imaging of articular cartilage morphology in the knee, and will specifically deal with different cartilage morphology outcome variables and regions of interest, the relative performance and relationship between cartilage morphology measures, reference values for MRI-based knee cartilage morphometry, imaging protocols for measurement of cartilage morphology (including those used in the Osteoarthritis Initiative), sensitivity to change observed in knee OA, spatial patterns of cartilage loss as derived by subregional analysis, comparison of MRI changes with radiographic changes, risk factors of MRI-based cartilage loss in knee OA, the correlation of MRI-based cartilage loss with clinical outcomes, treatment response in knee OA, and future directions of the field.

## 1. Introduction

**Figure 1:**
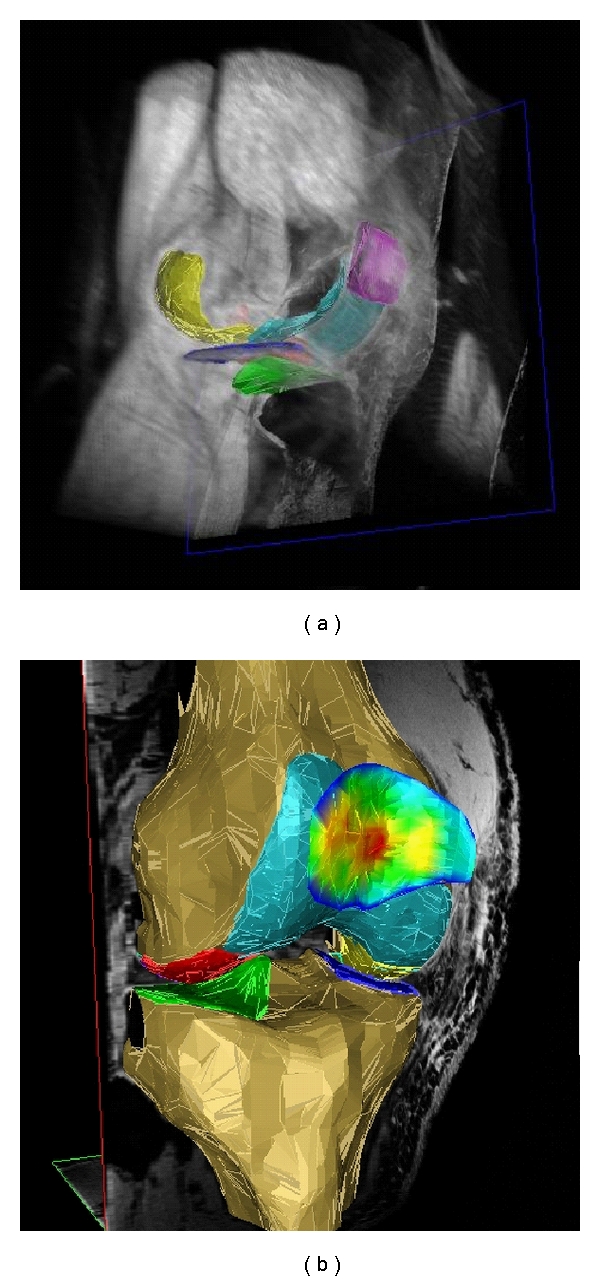
3D reconstruction of the knee cartilages after segmentation: (a) View from anteromedial with softtissues in grey (b) View from anterior-lateral, with the bone segmented and with the cartilage thickness distribution in the patella displayed in false colors (red: thick cartilage; blue: thin cartilage). The cartilage of the medial tibia (MT) is depicted dark blue, that of the lateral tibia (LT) green), that of the medial weight-bearing femoral condyles (cMF) yellow, that of the lateral weight-bearing femoral condyles (cLF) red, that of the patella (P) magenta, and that of the femoral trochlea (TrF) turquoise. Segmentation was performed based on a 3D-DESS knee imaging data set from the Osteoarthritis Initiative (OAI), a public-private partnership funded by the National Institutes of Health and conducted by the OAI Study Investigators. For anatomical (region of interest) labels, also see Figure 2 and Table 1.

Magnetic resonance imaging (MRI) has revolutionized the field of clinical research in osteoarthritis (OA) because it can directly visualize all diarthrodial tissues, including cartilage, bone, menisci, ligaments, synovium, and others. As it has been recognized that OA is a disease of the entire joint, involving most (if not all) of the above tissues, MRI has substantial advantages over radiography, which can only delineate the bone. Owing to its three-dimensional coverage of anatomical structures [1, 2] (Figure 1), MRI additionally permits to obtain quantitative measures of relevant tissue structures (and their changes over time) in OA. Quantitative measures of cartilage morphology (i.e., thickness, volume, surface areas) represent potentially powerful surrogate endpoints in osteoarthritis (OA). These can be used to identify risk factors of structural disease progression and can facilitate the clinical efficacy testing of disease (or structure) modifying drugs in OA (DMOADs), which are not clinically available to date.

This paper will focus on the knee, as most of the quantitative cartilage imaging work has been performed in that joint. It will further focus on the cartilage, as this the tissue that has generated most interest in context of quantitative measurement in OA using MRI. Last, we will focus on quantitative cartilage morphology (i.e., thickness, surface areas, volume) but will not cover quantitative MRI techniques measuring cartilage composition, such as dGEMRIC, T2, T1rho, and others [3]. 

Quantitative measurements of cartilage morphology (structure) fully exploit the 3D nature of MRI data sets [1, 2]; their strength is that they are less observerdependent and more objective than scoring methods, and that relatively small changes in cartilage thickness, which occur relatively homogeneously over larger areas may be detected over time, which are not apparent to the naked eye. This is important, as the progression of structural changes in OA has generally been shown to be slow, both when being evaluated by radiography [4–6] and MRI [6–10]. A recent study found that quantitative measures of cartilage morphology [11] were more powerful in revealing relationships between local risk factors (meniscus damage and malalignment) and knee cartilage loss than a semi-quantitative approach using ordinal scales (i.e., whole organ MRI score) [12]. The disadvantage of quantitative measurement, however, is that it requires specialized software and is more time intensive because tissue boundaries need to be tracked (i.e., segmented) throughout large series of slices using trained technical personnel. Also, quantitative measurements are less sensitive to the occurrences of small focal changes within larger structures (i.e., cartilage lesions), which may be readily picked up by an expert reader, particularly if the location within the larger structure is variable from joint to joint. A recent study showed, for instance, that MRI-based semiquantitative scoring of cartilage status was able to differentiate between knees with and without early (i.e., Kellgren-Lawrence grade [KLG] 2) radiographic OA, whereas quantitative measures of cartilage morphology displayed no or little difference between healthy and KLG2 knees [13]. It thus depends on the context and on the specific research question, whether or not quantitative cartilage assessment is better suited as an outcome measures for a particular study than semi-quantitative measures. Ideally, both approaches should be used in complimentary rather than competing fashion in studies assessing either the status or the progression of OA.

Focusing on quantitative imaging of cartilage morphology in the knee, this paper will sequentially address

- different cartilage morphology outcome variables and regions of interest in the knee,
- the relative performance and relationship between cartilage morphology measures,

(iii) imaging protocols for measurement of cartilage morphology, including validation,

(iv) rates of change and sensitivity to change observed in knee OA.

(v) spatial patterns of cartilage loss in knee OA as derived by subregional analysis,

(vi) comparison of MRI changes with radiographic changes in knee OA,

(vii) risk factors of cartilage loss in the knee as identified by quantitative cartilage MR imaging,

(viii) the correlation of MRI-based cartilage loss with clinical outcomes, and treatment response in knee OA,

(xi) future directions of the field.

## 2. Cartilage Morphology Outcome Variablesand Regions of Interest in the Knee

**Figure 2:**
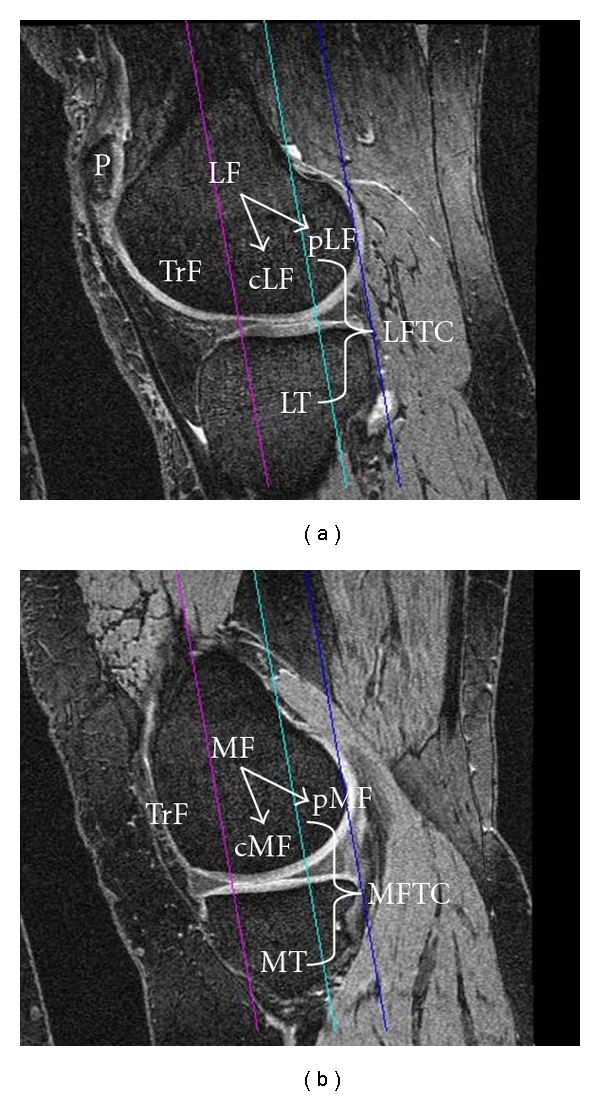
Sagittal 3D DESS MR images showing anatomical regions of interest commonly analyzed: (a) lateral femorotibial compartment, (b) medial femorotibial compartment; P: patella, TrF: femoral trochlear, MT: medial tibia, MF: medial femoral condyle, cMF: weight-bearing part of the medial femoral condyle, pMF: posterior part of the medial femoral condyle, MFTC: cMF + MT; LT: lateral tibia, LF: lateral femoral condyle, cLF: weight-bearing part of the lateral femoral condyle, pMF: posterior part of the lateral femoral condyle, LFTC: cLF + LT; the magental line shows the projection of the trochlear notch, the blue line the posterior end of the medial and lateral femoral condyle, and the turquoise line the 60% criterion (of the distance between the trochlear notch and the posterior ends of the condyles) used to separate cMF from pMF, and cLF from pLF, respectively. Images are from the Osteoarthritis Initiative (OAI), a public-private partnership funded by the National Institutes of Health and conducted by the OAI Study Investigators. For anatomical (region of interest) labels, also see Figure 1 and Table 1.

A consensus-based nomenclature for the above-mentioned structural (i.e., morphological metric labels) or compositional features as well as definitions for regions of interest in the knee (i.e., anatomical labels, see Table 1 and Figure 2) has been proposed by a group of experts [14]. The above nomenclature will be used throughout this paper, and important abbreviations for morphology metrics and anatomical regions of interests including recent extensions (i.e., statistical labels and subregional labels, i.e., [15]) are summarized in Table 1. Cartilage morphology outcomes commonly include the size of the total area of subchondral bone (tAB), the area of the cartilage surface (AC), the denuded (dAB) and cartilage covered (cAB) area of subchondral bone, the cartilage thickness over the tAB (ThCtAB) or over the cAB (ThCcAB), the cartilage volume (VC), the cartilage volume normalized to the tAB (VCtAB), the cartilage signal intensity [16–18], and others (Table 1).

To obtain the above quantitative morphological measures of cartilage, the relevant cartilage plates of a joint need to be segmented by a trained user with the choice of several input devices [19], with or without assistance from (semiautomated) segmentation software [20–28]. Because the relative performance of different segmentation algorithms has been discussed in previous reviews [9], this point will not be covered in depth in this chapter. Using the above tools, an operator needs to accurately trace both the bone-cartilage interface (i.e., the subchondral bone surface; tAB), and the surface of the cartilage, respectively (AC). The tracing of the tAB should include dABs, but not (peripheral) osteophyte surfaces. As there are various sources of artifacts on MRI, and because signal intensity and contrast may vary substantially between baseline and followup acquisitions, there exists a current consensus that expert quality control is important for accurate analyses; the time required for segmentation of the cartilages or for the correction of computer-generated segmentation may take several hours per knee joint. After all slices of interest have been segmented, image analysis software can be used to compute the three-dimensional morphological features listed in Table 1.

Anatomical regions of interest in the knee are listed and explained in Table 1 and Figure 2. Since the weight-bearing (cMF, cLF) and posterior aspects (pMF and pLF) of the femoral condyles are continuous and lack a definite anatomical border, different definitions for these ROIs have been proposed: Glaser et al. [32] used the projection of the posterior intercondylar bone bridge as a cutoff between the weight-bearing and posterior zone, whereas later studies [33] introduced a 60% distance criterion between the trochlear notch and the most posterior aspects of both femoral condyles as a cutoff between both regions (Figure 2). In a face-to-face comparison, the work in [34] reported the tAB of the 60% ROI to be approximately 20% greater and less variable (between subjects) than that based on the bone bridge, with cartilage morphology metrics being generally more reproducible in the 60% ROI. However, thickness measures did not differ significantly between both ROIs, and the longitudinal rate of change and standardized response mean (SRM = mean change/SD of change, as a measure of sensitivity to change) over two years were similar for both ROIs. Using sagittal images, both 60% [33] (Figure 2) and 75% [35, 36] cutoffs have been used, the 60% cutoff assigning 36 (±1.8%) of the tAB of MF to the weight bearing (cMF) and 64% to the posterior portion (pMF), whereas the 75% cutoff assigns 47% (±2.0%) of the tAB to cMF and 53% to pMF, respectively [36]. Again, the mean change and SRM were similar for both the 60% and the 75% ROI [36]. The (weight-bearing) medial femorotibial compartment (MT + cMF) is commonly addressed as MFTC, and the lateral compartment (LT + cLF) as LFTC (Figure 2).

Quantitative measures of surface curvature and joint incongruity have also been determined from MR images [37] and were observed to discriminate between subjects with various radiographic OA grades cross-sectionally at 0.2 T [38, 39]. Curvature estimates at different scales (at 0.2 T) were reported to be associated with the magnitude of cartilage loss longitudinally [40] and cartilage homogeneity (quantified by measuring entropy from the distribution of signal intensities in tibial cartilage from 0.2 T gradient echo images) was reported to discriminate between subjects without and with early radiographic OA [18]. This measure was proposed to be particularly sensitive in peripheral regions, where the cartilage is covered by the meniscus [41]. These results are surprising because other MRI techniques that have been validated for targeting specific macromolecules of the cartilage, such as collagen, proteoglycans, or water (T2 mapping, T1rho, dGEMRIC and others) [3, 42] have often been unsuccessful in discriminating between healthy knees and knees with early OA, and they have generally not been able to discriminate between different radiographic OA stages, in particular between early (preradiographic) OA and radiographic OA [6, 8, 43].

## 3. Relative Performance and Relationshipbetween Cartilage Morphology Measures

Most investigations dealing quantitatively with cartilage morphology in OA have focused on the cartilage volume (VC), but this outcome measure has a number of pitfalls. The ability to discriminate between OA and healthy subjects is limited, because cartilage volume is largely determined by bone size, which increases the intersubject variability and thus limits the ability to discriminate between subjects with and without cartilage loss [44]. This has led to misinterpretations in the literature, where it has been suggested that a high VC may be protective of OA, because men show higher VCs than women, and women are more susceptible to knee OA than men. However, men have mainly larger joint surfaces than women (and hence also larger VC) [45] even after adjustment for body height and weight [46]; VC should therefore not be directly compared between sexes. In longitudinal studies, the subchondral bone area has been shown to increase with aging, both in healthy reference subjects and in OA patients [47–49]. Such effects may mask a reduction in cartilage thickness in OA when measuring VC, because of the simultaneous expansion of the bone and cartilage layer. Therefore, alternative outcomes have been used, such as the VC normalized to the subchondral bone area (VCtAB), or the cartilage thickness over the entire subchondral bone area (ThCtAB) [44, 50]. 

In a recent study, Hudelmaier et al. [34] examined the relationship of the above parameters and their test-retest precision (at 3 T) in a set of 33 subjects, both without and with signs of radiographic osteoarthritis (reproducibility study). Further, they compared these parameters at baseline and at 2-year followup in 28 subjects with advanced radiographic osteoarthritis (sensitivity study). They found that the AC was larger than the tAB in all cartilage plates. In MT and LT, the cartilage volume divided by the total bone area (VCtAB) was similar to the mean cartilage thickness over the total bone area (ThCtAB.aMe), whereas in cMF and cLF the VCtAB was somewhat greater than the ThCtAB.aMe. Different implementations of measuring the cartilage thickness (e.g., minimal distance from bone to cartilage, or minimal distance from cartilage to bone, or the average of both) produced very similar values in all cartilage plates. The maximal thickness over the total bone area (ThCtAB.Max) was found to be almost twice as high as the mean thickness (ThCtAB.Me) in the femorotibial plates. Reproducibility errors for cartilage volume divided by the tAB (VCtAB) were similar to those for the cartilage thickness over the total bone area (ThCtAB) and tended to be smaller than those for cartilage volume (VC). The reproducibility errors were also similar for different implementations of the thickness measurements (see above). The maximal thickness over the total bone area (ThCtAB.Max) and the average of the top 1% greatest thickness values (ThCtAB.Mav) displayed larger reproducibility errors than the averaged mean cartilage thickness over the total bone area (ThCtAB.Me) in all cartilage plates, but reproducibility errors for ThCtAB.Mav tended to be smaller than those for ThCtAB.Max. In terms of the rate of (and sensitivity to) change, the cartilage volume divided by the total bone area (VCtAB), and the mean cartilage thickness over the total bone area (ThCtAB.aMe) exhibited higher rates of change and greater SRMs (greater sensitivity to change) than cartilage volume (VC) in MT, but the difference was only marginal in cMF. The rates of change and SRMs for cartilage thickness over the covered bone area (ThCcAB) tended to be less than for cartilage thickness over the total bone area (ThCtAB) and for cartilage volume (VC), independent of the specific implementation, but tended to be greater than those for cartilage surface area (AC) and the cartilage covered bone area (cAB). ThCtAB.Max and ThCtAB.Mav showed low rates of change and SRMs, in particular in cMF.  Table 2 lists the percent change, the SRM, the significance level, and the precision error (test-retest) in MT and cMF (60% ROI) for different morphological variables from this study [34]. In summary, the normalized cartilage volume (VCtAB) and the mean cartilage thickness over the entire subchondral bone area (ThCtAB.Me) tended to be more reproducible and more sensitive to change (SRM up to −0.62) than cartilage volume (SRM up to −0.44), cartilage thickness over the cartilaginous area (ThCcAB; SRM up to −0.48) or maximal cartilage thickness (SRM up to −0.35) [34].

Other publications also reported that the sensitivity to change for ThCtAB or VCtAB was greater than for VC [30, 31], whereas others found comparable SRMs for these variables [51, 52]. A recent paper [31] reported that, when cartilage loss was rapid (due to high mechanical challenge in mal-aligned knees), “horizontal” cartilage loss (i.e., an increase in denuded area = dAB) made a stronger contribution to the total cartilage loss (= reduction in ThCtAB), whereas when cartilage loss was relatively slow in neutrally aligned knees, the “vertical” cartilage loss (reduction ThCcAB) made a stronger contribution. This finding will need to be confirmed in other cohorts and pathos-phyiological conditions. Wirth et al. [53] recently explored the rate and sensitivity to change of the minimal cartilage thickness (ThCtAB.Min) and applied the measurement to central subregions of MT, LT, cMF, and cLF, respectively. In 156 participants of the Osteoarthritis Initiative (OAI), they found the one-year rate of the ThCtAB.Min changes to be greater than those of ThCtAB.Me, but also reported a greater standard deviation, so that ThCtAB.Min was found to be less sensitive to change than ThCtAB.Me.

A recent paper [54] investigated the mathematical relationship between the above morphologic measurements and explored whether a subset of the above variables fully reflects differences observed in cartilage in cross-sectional and longitudinal studies. The benefits of this reduction in variables are an increased statistical power due to less multiple comparison issues, an improved understanding of relationships between the morphologic measures of knee cartilage, and a greater efficiency in reporting the results in the literature. Buck et al. [54] used cross-sectional [55] and longitudinal (baseline to 2 year followup) 3T MR image data [29] from 152 women (77 healthy and 75 with knee OA). They found that the total area of the subchondral bone (tAB), cartilage thickness (ThCtAB.tAB), and the percentage of denuded area of the subchondral bone (%dAB) explained more than 90% of the cross-sectional and longitudinal variation in the full set of cartilage morphology measures, both in healthy and in osteoarthritic knees. The authors therefore recommended these three variables as an efficient subset for describing structural status and change in knee cartilage [54].

## 4. Reference Values for MRI-Based KneeCartilage Morphometry

Several groups have reported reference values of cartilage morphology in healthy volunteers [44, 56, 57], including templates/atlases for comparison of cartilage thickness distribution patterns between healthy reference subjects and OA patients [50, 58] and reference values for the radiographic joint space width (JSW) [59]. Beattie et al. [59] found that measures of JSW did not significantly decrease with increasing decade, but remained fairly constant throughout the lifespan in either sex; the same was observed for cartilage morphometry measures. The authors suggested that there may therefore be no need to differentiate a T- or Z-score in OA diagnosis because cartilage thickness and JSW remain constant throughout life in the absence of OA. 

Recently, several authors have proposed the measurement of certain anatomically defined subregions within cartilage plates to determine the spatial pattern of cartilage loss [15, 60, 61] (Table 1). A recent analysis of a large population-based cohort reported sex-specific normal values and potential maximal Z-scores for specific subregions of the femorotibial cartilage [62]. The authors studied 686 Framingham participants (309 men, 377 women, age 62 ± 8 years) without radiographic femorotibial OA (“normals”) and a subset of 376 Framingham participants (156 men, 220 women) who additionally had no MRI features of cartilage lesions (“supernormals”). The Framingham participants had thinner cartilage in the medial (3.59 mm) than in the lateral femorotibial compartment (3.86 mm). Medially, the femur displayed thicker cartilage (1.86 mm) than the tibia (1.73 mm), and laterally the tibia thicker cartilage (2.09 mm) than the femur (1.77 mm). The thickest cartilage was observed in central, and the thinnest in external femoro-tibial subregions. The mean values in Framingham “supernormals” and in non-exposed Osteoarthritis Initiative (http://www.oai.ucsf.edu/) reference participants (participants without symptoms or risk factors of knee osteoarthritis (OA)) were very similar to those in Framingham “normals”. The authors concluded that adequate reference values could be obtained from populations without radiographic OA (independent of risk factors and their specific MRI lesion status), and that a cartilage thickness loss of approximately 27% is required for attaining a Z-score of −2.

## 5. Imaging Protocols for Measurement ofCartilage Morphology Including Validation

Quantitative work performed on cartilage with MRI between 1994 and 2006 has been summarized previously [8, 9, 63] and will not be repeated in this paper. Briefly, for quantifying cartilage morphology, water-excitation (or fat-suppressed) T1-weighted spoiled gradient recalled echo acquisition in the steady state (SPGR) or fast low angle shot (FLASH) at 1.5 T or 3 T represent the current gold standard [9, 64, 65] for quantitative cartilage imaging. Double-echo steady-state imaging (DESS) with water excitation has recently gained interest because of the faster acquisition time and lower slice thickness that can be achieved (Figure 2) [33, 36, 66–68]. SPGR/FLASH sequences are readily available on almost all MRI scanners and do not require specific hard- or software, whereas the DESS is currently only available from one vendor [33]. Because the DESS acquires two separate images with different echo times simultaneously, this additionally provides potential opportunity to estimate T2 and to obtain morphological and compositional information of the cartilage from a single high-resolution data set [69]. This approach is still undergoing validation.

**Figure 3:**
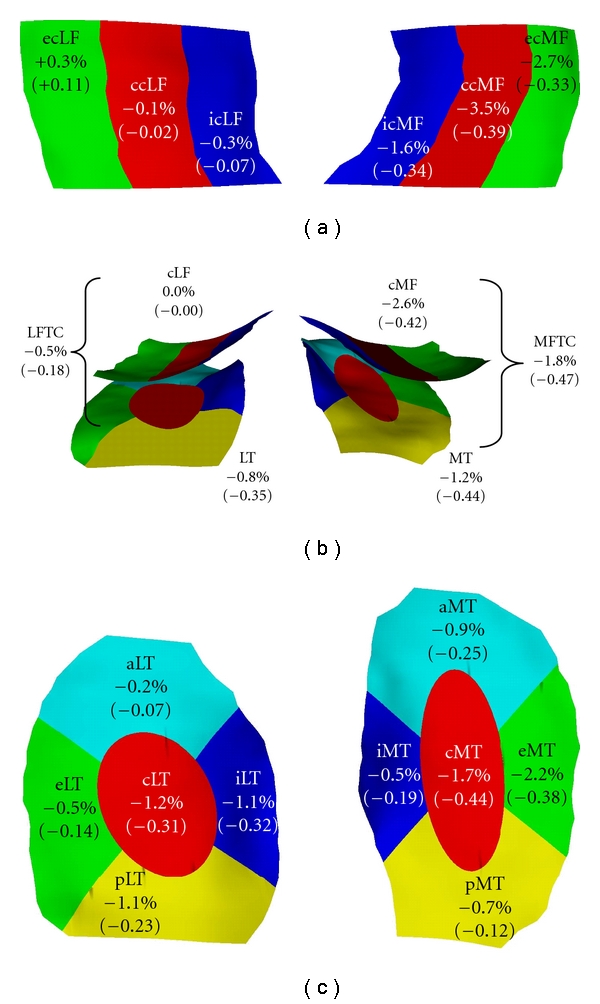
Display of the rates of change (%/annum) and standardized response mean (SRM) in femorotibial cartilage compartments, plates and subregions. (a) View of the weight-bearing part of the medial (cMF) and lateral femoral condyle (cLF) from inferior. (b) View of the weight-bearing part of the cMF and cLF and of the medial (MT) and lateral tibia (LT) from posterior. (c) View of the MT and LT from superior. For an explanation of the subregion abbreviations, please see Table 1. The data represent mean values from 3 studies: (i) the KLG3 participants of the A 9001140 study (*n* = 28) [29], (ii) the high risk (BMI > 30; KLG ≥ 2) subcohort from a first release of OAI participants (*n* = 54) [30], (iii) knees with neutral alignment from the MAK study (*n* = 74) [31].

The previously mentioned Osteoarthritis Initiative (OAI) (http://www.oai.ucsf.edu/) is a large research endeavor jointly sponsored by the National Institute of Health (NIH), the National Institute of Arthritis and Musculoskeletal and Skin Diseases (NIAMS), and the pharmaceutical industry. This study in a cohort of 4800 participants is currently focusing on identifying imaging (and other) biomarkers for predicting and monitoring the onset and progression of symptomatic knee OA using-3 T MRI over a 4-year period (currently being extended to 8-year followup). The OAI relies on a nearly isotropic sagittal DESS sequence with water excitation in both knees for quantifying cartilage morphology and on a coronal FLASH sequence with water excitation in one knee of all participants [70]. Sagittal images have the advantage that all cartilage plates of the knee (including the femoropatellar and femoro-tibial compartment) are visualized, but suffer from partial volume effects in the internal and external femoro-tibial subregions (Figure 3). Coronal images, in contrast, can delineate the femoro-tibial joint and axial images visualize the patella with little partial volume effects, but there is currently no consensus, which of the above is the preferred orientation. A direct face-to-face comparison of 2 year changes measured in coronal and sagittal (SPGR) images revealed similar rates and patterns of cartilage loss in the femoro-tibial joint [71]. 

The technical accuracy (validity) and test-retest precision (reproducibility) of quantitative cartilage measurements at 1.5 T have been summarized in previous reviews [8, 9]. Analyses based on 1.0 T images acquired with a dedicated extremity scanner were found to be consistent with 1.5 T imaging, albeit less precise (reproducible) [72]. The use of peripheral MRI scanners at lower field strengths potentially permits more widespread distribution of this technology, especially when access to high-field MRI is limited. Quantitative cartilage measurement at 0.2 T have also been proposed [18, 27, 28, 38–40] but have not been validated versus external standards or measurement at higher field strength. However, they were shown to display substantially larger precision errors than measurements performed at higher field strength. 3 T cartilage imaging has been cross-calibrated with 1.5 T and lower precision errors than for 1.5 T imaging were reported when acquiring thinner (coronal) slices of 1.0 mm on a 3 T system [73]. Morphometric analysis from DESS images, as acquired at 3 T in the OAI, was found to be consistent with that from FLASH images and to display similar test-retest precision errors as FLASH in the femoro-tibial joint, both using unpaired [33] and paired reading approaches [67, 68]. In terms of sensitivity to change, Wirth et al. [36] performed a face-to-face comparison between FLASH and DESS over one year longitudinally in 80 knees. The study confirmed a high agreement between cartilage thickness measures as determined from FLASH and DESS cross-sectionally [33] and a similar sensitivity to change of coronal FLASH and sagittal DESS. Further, the study revealed a moderate correlation of the longitudinal one-year changes, indicating that it may be adequate to pool analyses obtained with FLASH and DESS in larger statistical analyses [36]. Also, the authors found that analysis of every 2nd slice (i.e., obtaining information every 1.4 mm) of the sagittal DESS displayed similar SRMs as compared with segmentation of every 0.7 mm slice, both when either using odd or even slice numbers [36]. Due to the near-isotropic resolution of the sagittal DESS, multiplanar reconstruction (MPR) in the coronal and axial planes is feasible [33, 67, 68]. The rates of (and sensitivity to) change of coronal MPR DESS was similar to that of coronal FLASH and sagittal DESS but did not provide an advantage over the direct analysis of the sagittal DESS [36].

Generally, results from different vendors for cartilage morphometry were shown to be comparable at 1.5 T [74] and at 3 T [75], although one study reported slight offsets between different scanners and protocols from the same vendor [76]. At 3 T, precision errors of cartilage morphometry were observed to be similar for different vendors and scanners in a multicenter trial, and measurements were relatively stable over a 3-month observation period [48]. The stability of geometric measurements over longer periods on phantoms was found to be satisfactory and comparable between several scanners of the same manufacturer over a 3-year period in the OAI [77].

Use of different coils has been evaluated at 3 T. Although the test-retest precision was similar between a phased array and quadrature coil, certain offsets in cartilage morphology outcomes were observed [67]; these prohibit changes of the coil between baseline and followup measurements. Cartilage morphometry on images acquired 2 hours after intravenous Gd-DTPA injection (for the purpose of simultaneous dGEMRIC imaging) was reported to be highly correlated (*r* = 0.85 − 0.95) with that on images obtained before the injection of the contrast agent at baseline [78]. However, a 2-year longitudinal analysis in OA participants reported that the sensitivity to change of post-Gd-DTPA cartilage imaging was substantially less than that from images acquired prior to intravenous Gd-DTPA injection [79].

## 6. Rates of Change and Sensitivity to Change Observed in Knee OA

Numerous reports on longitudinal changes of cartilage morphology in subjects with different grades of knee OA have been published [8, 9, 29–31, 36, 52, 53, 61, 80–87]. These studies have revealed variableresults with regard to the rates of cartilage loss and SRM [6, 8, 9] Two studies reported almost no loss in cartilage volume over a 1-year [87] and 3-year period [80], respectively, whereas other studies reported up to 7% annual cartilage loss in the femoro-tibial joint [82]. Reasons for this may include variability in imaging and image analysis technology, differences in risk factor profiles between cohorts, differences in study duration, experience and blinding of the readers, and others. A recent study [88] tested the hypothesis that “Proof of Concept” studies with shorter durations may be achievable with 3 T MRI, by selecting populations at high risk of rapid medial femoro-tibial progression and using advanced image analysis techniques. Female participants with knee pain, a body mass index ≥ 25, and radiographic evidence of medial OA and varus mal-alignment were monitored over 3 and 6 months, respectively, and anatomically corresponding ROIs were identified on each image by using a three-dimensional statistical shape model of the bone surface. The primary outcome was the change in cartilage thickness in the aspect of cMF that is exposed within the meniscus window during articulation, excluding the peripheral aspects of the femoral surface. Despite these efforts, no change in ThCtAB was detected at *P* < .05 at 3 or 6 months followup; the mean change at 3 months from a log-scale ANOVA model was −2.1% [95% confidence interval (CI) (−4.4%, +0.2%)] and the change over 6 months was 0.0% [95% CI (−2.7%, +2.8%)]. Changes in the lateral tibia were significant at 6 month followup (−1.5%), but only without correction for multiple comparisons. The authors concluded that the small inconsistent compartment changes and the relatively high variability in cartilage thickness changes seen in the study provided no confidence for a 3- or 6-month study, not even based on a patient population selected for rapid progression [88].

Analyses of the first release of 160 participants of the OAI progression cohort (baseline and year 1 followup data) found significant change of up to 2% per annum, with substantially higher rates of progression in the cMF than in the MT, and higher rates in LT than in cLF [30, 52, 53]. However, this pattern of change was not entirely consistent across cohorts, when focusing on the SRM rather than on the rate of change [10, 29, 31]. Several studies therefore have taken the approach of reporting the aggregate thickness in the tibia and weight-bearing femur (MFTC or LFTC) [29–31, 36, 67, 89]. One study suggested that longitudinal changes in VC in the tibia and in the weight-bearing femur are highly correlated [82], and that the measurement of only tibial cartilage is therefore sufficient. However, given that at least some cohorts appear to display larger changes and higher SRMs in the weight-bearing femur than in the tibia [30, 52, 53], this approach has limitations. 

Medial and lateral femoro-tibial cartilage loss as well as patellar cartilage loss were found to be not significantly associated with each other [90]. The ratio of medial versus lateral cartilage loss was reported to be 1.4 : 1 in knees with neutral biomechanical alignment, consistent with higher mechanical loads being transferred across the medial compartment in neutral knees [31]. In varus knees, the ratio was 3.7 : 1, and in valgus knees it was 1 : 6.0, confirming that knee alignment is an important determinant of medial versus lateral rates of cartilage loss [31]. 

After anterior cruciate ligament rupture, a reduction of cartilage volume and thickness was observed in the femoral trochlea (TrF), while an increase was found in the weight-bearing medial femur (cMF) [91]. The latter observation may be consistent with cartilage swelling or hypertrophy observed as a sign of early OA in various animal models [92–96]. A recent cross-sectional study found significantly thicker cartilage in the medial compartment of women with medial radiographic KLG2 OA compared with healthy knees [55] and significantly thinner cartilage in some subregions in knees with medial radiographic OA with joint space narrowing (JSN, i.e., KLG3). These observations were confirmed by a large cross-sectional analysis of more than 1000 OA participants, in which the authors [97] confirmed a significantly greater cartilage thickness in KLG2 compared to healthy knees, specifically in the external subregion of the medial femur (ecMF), both in men and in women. These findings have suggested that there may be an initial phase of cartilage swelling/hypertrophy in knee OA, particularly at the KLG2 stage, which is characterized by osteophytes without a reduction in JSW. This has been supported by recent longitudinal observations by Buck et al. [98] who explored whether the 2-year longitudinal change in cartilage thickness in femoro-tibial subregions (see below) of knees with radiographic osteoarthritis (ROA) differed from that in healthy knees. Knees from 75 women with definite signs of medial radiographic OA were compared with 77 asymptomatic healthy controls without radiographic OA. A substantial portion of ROA knees were classified as having longitudinal cartilage thinning (28%) or thickening (20%) in at least one medial femoro-tibial subregion compared with longitudinal changes in healthy knees, and only 5% showed both subregional thinning and thickening at the same time, across (different) medial subregions. Whereas the estimated proportion of KLG3 knees with significant medial cartilage thinning (46%) was substantially greater than that with cartilage thickening (18%), the estimated percentages of KLG2 knees with significant medial thinning (20%) and thickening (23%) were similar. The subregion in which cartilage thickening was observed was ecMF in the majority of the cases. The authors concluded that OA may not be a one-way road of cartilage loss and that particularly in early radiographic OA, cartilage changes may occur in both directions simultaneously, that is, cartilage thinning and cartilage thickening. This may provide a reason why relatively small (and variable) rates of change have been observed in OA cohorts, and why short-term trials are challenging [88].

## 7. Spatial Patterns of Cartilage Loss in Knee OA as Derived by Subregional Analysis

As mentioned above, recent efforts have been focused on measuring anatomically defined subregions within cartilages [15, 60, 61], with the aim of elucidating spatial pattern of cartilage thinning, and to potentially identify (sub) regions with increased rates of (and sensitivity to) cartilage loss in intervention trials.

### 7.1. Cross-Sectional Studies

The previously mentioned cross-sectional study by Hellio Le Graverand et al. [55] reported that cartilage “thinning” in female knees with medial JSN (KLG3) was most evident in the central subregion of the cMF and in the external subregion of the MT, and in the internal subregion of the LT. This was extended by the study of Frobell et al. [97], who reported that the external medial tibia showed the greatest reduction in cartilage thickness (z-scores −5.1/−5.6 in men/women) in knees with medial joint space narrowing (OARSI JSN) grade 3 and the external lateral tibia (z scores −6.0 for both sexes) the greatest reduction in knees with lateral JSN grade 3. The authors, however, reported that at least 25% of the average normal cartilage thickness was maintained in all subregions of end-stage ROA knees. 

Although these differences were generally not affected when possible effects of demographic covariates (height and BMI) were considered [55, 97], it is difficult to exclude confounding by interperson differences in cross-sectional studies. Therefore, Eckstein et al. [99] performed a within-person, between-knee comparison in 80 participants of the OAI who displayed medial JSN in one knee, but no medial or lateral JSN in the contralateral knee. The strength of this approach is that it rules out confounding from person-specific demographic features, and that it is potentially more sensitive to detecting differences cross-sectionally, given the much smaller magnitude of side differences between knees within the same (healthy) person compared with differences across (healthy) subjects [100]. The authors estimated the magnitude of cartilage thickness reductions to be 190 *μ*m (5.2%) in the medial femoro-tibial compartment (MFTC) with JSN OARSI grade 1, 630 *μ*m (18%) with OARSI grade 2, and 1560 *μ*m (44%) with OARSI grade 3 [101, 102]. Side differences were greater in cMF than in MT, and greater in MT than in pMT [99]. Within MT the greatest differences were observed in the external and central subregions, and within MF the greatest differences were observed in the central subregion of the weight-bearing portion of MF. When evaluating A-P subregions in the MF [103], the greatest differences between mJSN and contra-lateral no-mJSN knees were observed in regions located between 30° and 75° at the MF.

### 7.2. Longitudinal Studies

Pelletier et al. [61] reported that the rate of change in cartilage morphology in the central aspects of the femoro-tibial joint exceeded that in total cartilage plates, but found that the SRM was not improved because of the higher variability of subregional changes [51]. Wirth et al. [53] found the sensitivity to change (SRM) in the central MT to be slightly greater than for the total MT in a subsample of OA Initiative participants, but this finding was not confirmed in the cMF.

Figure 3 summarizes the rates of change (%/annum) and the sensitivity to change (SRM) for different subregions from three published studies.

- A 2-year multicenter study at 3T (Pfizer A 9001140). Because healthy reference participants and participants with KLG 2 did not show significant changes in cartilage morphology [29], results from KLG 3 participants were used (*n* = 28).

(b) The 2nd cohort included is a first release of baseline and year 1 followup data from the OAI progression subcohort [53]. Results of a subcohort with a high risk of progression (BMI > 30; KLG ≥ 2) were included (*n* = 54).

(c) The 3rd cohort included was from the MAK study [11, 31]. Data from a subcohort of participants with neutral knee alignment were included in the analysis (*n* = 74).

The central and external part of cMF, and the external and central aspect of MT displayed the relatively greatest change across subregions in the MFTC (Figure 3). With the exception of the external medial femur, these regions consistently displayed greater changes than the total cartilage plate across the studies. In the LFTC, the central, internal, and posterior LT displayed the relatively greatest changes, and no relevant average changes (across studies) were observed in the cLF (Figure 3). Rates of change in the central and internal LT were consistently greater than those for the total cartilage plates. Please note that the patterns for the sensitivity to change (SRM; Figure 3) are similar to those of the rates of change, but not identical. Consistent with other observations in the literature [51], we found that the sensitivity to change in the subregions was not consistently higher than in the total plates across the above three studies. However, analysis of the central aspects (subregions) of the medial and lateral femoro-tibial compartments revealed consistently greater SRMs than the analysis of the entire MFTC and LFTC, respectively.

Wirth et al. [103] recently presented a method which extended the previously developed method of subregions in the weight-bearing femoro-tibial joint [15] to anterior-posteriorly spaced subregions across the entire femoral condyle. This method was applied to participants from the OAI and confirmed that cartilage thinning in the anterior (weight-bearing) region of the MF was greater than that in the posterior aspect of the MF. The authors reported the greatest longitudinal changes (and SRM) to be located at 30 to 60° (from the trochlear notch [0°] to the posterior/superior end of the MF (150°), with a slight variation between knees with different OARSI JSN grades.

### 7.3. Ordered Value Approach (Subregion Ranking)

Buck et al. [104] analyzed patterns of subregional cartilage change [15] in individual knees and found highly variable patterns of change. To compare the rate of change between two groups (i.e., ROA knees with healthy knees, or DMOAD treated knees with control knees) he therefore recommended the use of ordered values (OVs) or ranking system, in which the subregional changes (in MFTC) were assigned to ranked orders in each knee, that is, the subregion with greatest magnitude of cartilage thinning to OV1, the one with the second greatest magnitude to OV2, and the one with the smallest magnitude of cartilage thinning (or with the greatest magnitude of cartilage thickening) to the highest rank order. When averaging longitudinal changes in cartilage thickness (ThCtAB) across these OVs (which vary in location across subjects), the authors found that the minimal *P* value (Wilcoxon) for the differences in 2-year change in medial cartilage thickness in a relatively small number of knees with radiographic OA and JSN (KLG3) versus healthy knees (KLG0) was *P* = .001, with OV1 to OV4 displaying significant differences between both groups. When averaging changes across compartments, plates, or subregions (i.e., the conventional approach), in contrast, only one medial subregion displayed significant differences (in the rate of change) between KLG 3 and KLG0 knees (*P* = .037). Cartilage thickening was significantly greater in knees with radiographic OA (definite osteophytes) without JSN (KLG2) versus KLG0 knees in one medial subregion using the conventional approach (*P* = .02), but in two OVs using the ordered values approach (minimal *P* = .007). The authors concluded that the ordered values approach was more sensitive in detecting cartilage thinning in KLG3 versus KLG0, and cartilage thickening in KLG2 versus KLG0 knees, respectively. The authors also suggested that this method was particularly useful in the context of comparing a cohort treated with a disease-modifying OA drug versus one treated with a placebo, or in detecting risk factors of OA progression. 

Wirth et al. [105] recently extended this approach to include eight medial and eight lateral (*n* = 16) subregions. They reported significantly greater cartilage loss in KLG3 than in KLG2 knees, the ordered value approach again displaying considerably smaller *P* values than the conventional approach. This opens new possibilities of including participants with medial and lateral OA (or with varus and valgus mal-alignment) into a study, without the need of defining cartilage thickness changes in a certain compartment, plate or subregion as the primary endpoint. The relevant question would then NOT be whether a certain risk factor is associated with or whether a drug can modify cartilage thickness changes in a given location (region), BUT whether the risk factor is associated with or whether the drug can modify the change in cartilage thickness wherever it occurs in an individual knee.

## 8. Comparison of MRI Changes withRadiographic Changes in Knee OA

Several studies found only weak correlations between MRI-detected cartilage loss and OA progression in radiography [83, 87, 106]. However, a recent publication reported a stronger correlation when the longitudinal reduction in JSW in radiographs was compared with cartilage loss in the central aspect of the MFTC [61]. Whereas some studies found a higher rate and sensitivity to change of MRI-based measurements of cartilage morphology compared with radiography [83, 85, 107], a recent analysis reported a somewhat greater SRM (−0.62) for fluoroscopy-based Lyon Schuss radiography versus ThCtAB of the MT measured with MRI (−0.59) [29]. However, the authors found the SRM for fixed flexion radiography, a commonly used nonfluoroscopic protocol that also is used in the OAI [108–112], to be substantially less (SRM = −0.20) in the same study [29]. The authors argued that the relatively high SRM of the minimal JSW measured by Lyon Schuss may be due to the fluoroscopic guidance providing optimal alignment of the anterior and posterior tibial rim, and to radiography being performed under weight-bearing conditions where the cartilage tissue is compressed, while MRI is performed in a supine non weight-bearing position. Also, it must be kept in mind that radiographic assessment of JSW depends also on meniscus extrusion, and not only on cartilage thickness [113–115] and that meniscus pathology, in particularly subluxation, can therefore cause changes in JSW over time in the absence of cartilage loss. Duryea et al. recently compared the responsiveness (=sensitivity to change) of radiography with that of MRI in the first release of the OAI cohort (150 subjects) over 12 months [116]. The radiographic JSW measurements relied on automated software to delineate the femoral and tibial margins [117, 118]. Measures included the medial compartment minimum JSW and JSW at fixed locations that were compared to previously published cartilage morphology measures [52]. The SRM value for radiographic JSW measured at the optimal fixed location was −0.32 compared to −0.39 for the most responsive MRI measure. For a subsample of KLG2 or KLG3 knees, the most responsive SRM values were −0.34 and −0.42, respectively. The authors concluded that new (fixed distance) measures of JSW changes were superior to conventional minimal JSN measures and provide a similar sensitivity to change as MRI.

## 9. Risk Factors of Cartilage Loss in Knee OA as Identified by Quantitative Cartilage Imaging with MRI

Great interest is directed at identifying risk factors (predictors) of subsequent cartilage loss, both to understand the pathophysiology of the disease and to be able to identify so-called fast progressors for inclusion in pharmacological intervention studies that attempt to show protection from structural change over relatively short periods (e.g., [88]). This paragraph will focus on studies that have reported correlations between risk factors of progression and quantitative measures of cartilage morphology, but not those that have relied on semi-quantitative scoring of MRI or quantitative measurement of JSW. The list of (potential) risk factors for cartilage loss is not complete, but encompasses important examples examined both from cross-sectional and longitudinal studies. Risk factors associated with higher rates of progression were the following.

**Advanced Radiographic OA and Low Cartilage Thickness at Baseline**

Opposite to earlier assumptions and a synthesis of evidence from radiographic studies [119], recent evidence suggests that advanced radiographic OA (JSN) is a strong (if not the strongest) predictor of fast progression (i.e., cartilage loss). There has been evidence that knees with higher KL grades and increased JSN [29, 30, 53, 61] display greater rates of (and sensitivity to) change than those with lower KL grades and without baseline JSN. An analysis of specific radiographic features in a sample from the OAI found that osteophyte status (at baseline) was not associated with medial cartilage loss over 12 months but that knees with medial joint space narrowing showed a trend towards higher rates of change than those without, and that knees with medial femoral subchondral bone sclerosis displayed significantly greater rates of progression than those without [120]. The same study also found that low baseline cartilage thickness was a strong predictor of longitudinal loss in cartilage thickness [120], whereas an earlier study had reported that higher baseline cartilage volume [81] was strongly associated with increased cartilage loss. A within-person, between-knee comparison in painful knees selected from the OAI [35] recently reported that the cartilage loss was greater in knee with radiographic JSN than in contra-lateral knees without JSN in the same subjects, and that the side differences were greater with higher grades of JSN. Progression was particularly fast in the small subgroup with OARSI JSN grade 3 knees [35].

**Meniscus Extrusion and Tears/Damage [61, 84, 85]**

Meniscus tears were found to be associated with greater tibial plateau bone area, but not with reduced tibial cartilage volume in a two year longitudinal study [121]. However, Sharma et al. [11] reported a significant relationship of cartilage loss with meniscus tears, albeit not with meniscus extrusion. A recent analysis found site-specific relationships between local meniscus tears and subregional cartilage loss, suggesting that a tear in the anterior horn, central part, or posterior horn of the meniscus was associated with increased cartilage loss in adjacent tibial subregions [122]. Crema et al. [123] reported grade 2 and 3 medial meniscus lesions to be associated with greater cartilage loss in the femoro-tibial compartment, but not grade 1 lesions (=intrasubstance meniscus signal changes). They concluded that the protective function of the meniscus was preserved in case of these early lesions. Recent evidence suggests that the meniscus may undergo a phase of hypertrophy in OA [124, 125]. Raynauld et al. [126] observed that selecting a subcohort of participants with meniscus tears/extrusion did not improve the ability to identify treatment effects of a potentially structure modifying drug, because of the larger standard deviation of the change in the participants with meniscus pathology.

**Knee Malalignment and Adduction Moment**

A strong relationship was observed between (varus and valgus) mal-alignment and the ratio of cartilage loss in MFTC versus LFTC [11, 31, 127, 128]. After adjustment for meniscus changes, the study by Sharma et al. [11] found that varus malalignment and medial meniscus damage both predicted medial tibial cartilage volume (and thickness) loss. In contrast, medial-lateral joint laxity, measured with a device applying a fixed varus and valgus load, was not found to have consistent effects and was not a significant predictor of cartilage loss in models fully adjusted for alignment and meniscus damage [11]. Teichtahl et al. [129] showed an increase in varus mal-alignment between baseline and followup to be associated with an increase in the rate of MT cartilage loss, whereas there was no significant correlation with the rate of cartilage loss of the LT. The authors concluded that methods to reduce progression of varus alignment may also delay the progression of medial femoro-tibial OA. Frontal plane knee valgus mal-alignment was also correlated with patellar cartilage loss [130]. In a largely nonarthritic cohort, in contrast, no correlation between cartilage loss and mal-alignment was identified [131]. A recent cross-sectional analysis revealed that a higher peak knee adduction moment was observed in participants with medial compared to those with lateral meniscus tears [132]. Participants with a higher knee adduction moment displayed a larger medial meniscus extrusion and lower medial meniscus height, whereas the inverse relationship was observed for the lateral meniscus. A higher knee adduction moment was also associated with a higher ratio of the medial to lateral tibial subchondral bone area, whereas cartilage thickness and denuded areas in the tibia and femur were not related to the knee adduction moment. Similar results were found for the relationship between knee adduction angular impulse and meniscus, cartilage, and bone morphology [132].

**High BMI**

In contrast with a synthesis from the radiographic literature [119], MRI-based studies on progression have found higher rates of cartilage loss in subjects with a high BMI [30, 53, 61, 83, 85, 90, 133]. This relationship was also suggested to exist in the patella in subjects without OA [134].

**Bone Marrow Alterations [61, 85]**

Raynauld et al. [126] reported that although bone marrow lesions and cysts did not increase significantly in size over 24 months in an OA cohort, there was a significant correlation between size change of bone marrow lesions and cysts with the loss of cartilage volume in the medial femoro-tibial compartment. A relationship between very large bone marrow lesions and lateral tibial cartilage loss was also reported in asymptomatic persons [135, 136].

**Focal Cartilage Lesions or Defects as Graded by Visual Scoring and Denuded Areas as Determined Quantitatively from MRI**

Cartilage defects at baseline (visual scoring) appeared to be associated with longitudinal measurement of quantitative cartilage loss in the same compartment in OA subjects [137, 138], although the second of the two above studies [138] only found a significant relationship in the femoro-patellar but not in the femoro-tibial joint. Other studies reported that the presence of cartilage defects predicted knee cartilage loss also in asymptomatic individuals without radiographic knee OA [139, 140]. It was hypothesized that tibial subchondral bone area expansion may lead to the development of knee cartilage defects (which are associated with future cartilage loss) and is predictive of the need for knee joint replacement in subjects with knee OA, independent of radiographic change [141]. Morphometric studies have recently provided evidence that areas of denuded subchondral bone (dABs), as determined by segmentation at baseline [142], also predict subsequent cartilage loss [120, 143]. Hunter et al. [143] reported that in a subsample of knees with no denuded area (at baseline) the SRM for subsequent cartilage volume loss was −0.25, whereas it was −0.30 in the knees with intermediate denuded areas and −1.0 in knees in knees with severe denuded areas. Denuded areas were observed to either originate from cartilage loss or from internal osteophytes [142]. In a subsample from the OAI, almost half of the men and a third of the women displayed dABs; 61% of the dABs represented internal osteophytes. One of 47 knees with KLG0 displayed any dAB, whereas 29 of the 32 KLG4 knees were affected. There were significant relationships of dAB with increasing KL grades (*P* < .001) and with ipsi-compartimental JSN. Internal osteophytes were more frequent laterally (mainly posterior tibia and internal femur), whereas full thickness cartilage loss was more frequent medially (mainly external tibia and femur).

**Molecular Markers from Biological Fluids**

Dam et al. [144] reported a significant association between baseline levels of the C-terminal telopeptide of type II collagen (CTXII) and cartilage loss in 158 study participants (36 with ROA at baseline) using low field (0.2 T) MRI–based cartilage loss. In this study, elevated CTXII was also associated with radiographic progression (by KLG or JSN, but did not reach statistical significance [144]. Bruyere et al. [145] followed 62 patients with knee OA using 1.5 Tesla MRI and found that baseline cartilage oligomeric matrix protein (COMP), C-terminal telopeptide of type I collagen (CTXI), and CTXII did not correlate with one-year changes in cartilage thickness, but longitudinal increase in CTXII over three months did (*P* = .04). Pelletier et al. [146] reported higher baseline values of interleukin 6, C-reactive protein, and COMP to be predictive of greater cartilage loss with MRI, whereas Eckstein et al. reported a relatively large set (*n* = 16) of different molecular markers of bone formation, bone resorption, cartilage synthesis, cartilage degradation, and inflammation take at baseline to be substantially less predictive of cartilage loss than simple radiographic measures, such as reduced JSW, or low baseline cartilage thickness [147].

**Other Risk Factors**

Some evidence has been provided that smoking may be associated with increased cartilage loss [140, 148, 149] in line with previous radiographic studies, but other factors such as age, sex, pain, function, physical activity levels, synovitis (effusion), sex hormone levels, and serum or urine biomarkers were not consistently found to be associated with cartilage thinning measured quantitatively with MRI and studies (including those with radiography) have produced partially contradictory results [119].

## 10. The Correlation of MRI-Based Cartilage Loss with Clinical Outcomes and Treatment Response in Knee OA

Estimates of tibial cartilage loss over two years were suggested to be correlated with those over 4.5 years, albeit the authors did not report the consistency of the longitudinal changes in the second versus the first observation period [86]. More importantly, however, the rate of change in VC over 2 years was significantly associated with total knee arthroplasty (TKA) at year 4 [150]. For every 1% increase in the rate of cartilage loss there was a 20% increased risk of undergoing TKA and participants in the highest tertile of tibial cartilage loss had a 7.1 higher odds of TKA than those in the lowest tertile. In contrast, radiographic scores of OA did not predict TKA in the same study. A more recent study concluded from the same sample that when subchondral bone cysts were present, cartilage loss and risk of knee replacement were higher than if only bone marrow lesions were present, suggesting that cysts identify those that may benefit most from the prevention of structural disease progression [151]. These are important findings as they link longitudinal changes in cartilage morphology as a potential surrogate measure of disease progression, to a clinical outcome (i.e., how a patient feels or functions, or how long the knee “survives” [TKA]). 

Raynauld et al. [107] recently reported that licofelone (a drug that inhibits both cyclooxygenase and lipoxygenase) significantly reduced cartilage loss over time when averaged over both femoro-tibial compartment, and that MRI was superior to radiographs in demonstrating a structure modifying effect in this multicentre trial. Interestingly, the effects were significant only in the lateral, but not in the medial compartment, although the participants had been selected for medial femoro-tibial radiographic OA, and the medial compartment had thus been defined as the primary endpoint. To date, no structure or disease-modifying drug (SMOAD or DMOAD) has yet been approved by regulatory agencies, neither based on radiographic nor on MRI-based evidence of structure modification in knee cartilage.

## 11. Future Directions of the Field

In the Osteoarthritis Initiative, baseline, 12-, 24-, and 36-month followup clinical, radiographic, and MRI data have been made publicly available for approximately 4800 participants of the OAI cohort (http://www.niams.nih.gov/ne/oi/), and central readings of fixed flexion radiographs (OARSI atlas scores [101, 102]), quantitative measurements of radiographic joint space width (JSW) as well as quantitative cartilage morphology outcomes from MRI are available for various subsets that have been read/analyzed by expert reading facilities. These and the results of other large epidemiological studies will provide ample opportunity for collaborative research and should allow the research community to make rapid progress in understanding the risk factors involved in quantitative cartilage loss in OA. Most importantly, it will allow one to determine which imaging biomarkers can best predict clinical outcomes, such as real or virtual TKA [152]. This will be an important step in validating novel cartilage imaging biomarkers and approaches as surrogate measures of disease progression, particularly in therapeutic intervention trials. Once the clinical importance of these imaging biomarkers are established, further improvements in imaging hardware, coils, sequences, and image analysis algorithms may foster a more automated analysis of cartilage morphology, composition, and other articular tissues than currently possible. This will be of particular importance once structure- or disease-modifying drugs become available, as this may require monitoring the treatment response in large sets of OA patients. Currently, quantitative MRI of articular cartilage represents a powerful research tool in experimental, epidemiological, and pharmacological intervention studies. Once structure- or disease-modifying drugs (SMOADs or DMOADs) will become available, quantitative MRI of the cartilage may also play a more important role in clinical decision making and practice.

##  Disclosures

F. Eckstein is co-owner and CEO of Chondrometrics GmbH, a company that licenses software to academic researchers and provides image analysis service for academic researchers and the pharmaceutical industry. F. Eckstein provides consulting services to Merck Serono Inc. and Novartis Inc. and has received research funding from Pfizer, Eli Lilly, MerckSerono, Glaxo Smith Kline, Centocor Research, Wyeth, and Novartis. W. Wirth has a part time appointment with Chondrometrics GmbH.

## Figures and Tables

**Table 1:** Morphological (metrics), statistical, and anatomical (region of interest) labels commonly used in cartilage morphology publications on the knee.

Abbreviation	Explanation	Unit
*Morphological (metrics) label*		
VC	volume of the cartilage	(mm^3^/mL)
tAB	total area of subchondral bone	(cm^2^)
AC	area of cartilage surface	(cm^2^)
cAB	area of tAB covered by AC	(cm^2^)
dAB%	percent of tAB denuded (not covered by AC)	(%)
VCtAB	volume of the cartilage divided by tAB	(mm)
ThCtAB	thickness of the cartilage over the entire tAB	(mm)
ThCcAB	thickness of cartilage over cAB	(mm)

dAB%	percent of tAB denuded (not covered by AC)	(%)
VCtAB	volume of the cartilage divided by tAB	(mm)
ThCtAB	thickness of the cartilage over the entire tAB	(mm)
ThCcAB	thickness of cartilage over cAB	(mm)

*Statistical labels*		
Me	mean (i.e., thickness)	
Max	maximum (i.e., thickness)	
Mav	maximal averaged, for example, mean of the top 1% values	
Min	minimum (i.e., thickness)	
Miv	minimum averaged, for example, mean of the lowest 1% values	
SD	standard deviation (i.e., thickness)	
CV%	coefficient of variation (i.e., thickness)	
c(Me, Mav)	thickness measured from cartilage surface (AC) to bone interface (tAB)	
b(Me, Mav)	thickness measured from bone interface (tAB) to cartilage surface (AC)	
a(Me, Mav)	average of the two above (b, c)	

*Anatomical (region of interest) labels*		
Total cartilage plates		
P	Patella	
MT	Medial tibia	
LT	Lateral tibia	
F	Femur	
TrF	Femoral trochlea	
MF	Medial femoral condyle	
cMF	weight-bearing portion of MF	
pMF	posterior portion of MF	
LF	Lateral femoral condyle	
cLF	weight-bearing portion of LF	
pLF	posterior portion of LF	
MFTC	aggregate values for MT and cMF (MT + cMF)	
LFTC	aggregate values for LT and cLF (LT + cLF)	

*Subregions (to be combined with above total plate labels, i.e., cMT or ccMF)*		
c	central	
e	external	
i	internal	
a	anterior	
p	posterior	

For anatomical (region of interest) labels, also see Figures 1 and 2.

**Table 2:** Rate of change and sensitivity to change over 2 years in 28 participants with Kellgren-Lawrence grade [KLG] 3, and test-retest reproducibility in 33 participants with KLG0 to KLG3 for various cartilage morphology metrics and regions of interest in the medial femorotibial compartment.

	MT				cMF 60%			
	MC%	SRM	*P*	RMSCV%	MC%	SRM	*P*	RMSCV%
VC	−2.3	−0.44	.03	2.5%	−3.5	−0.32	.10	2.6%
tAB	0.5	0.37	.06	1.0%	−0.1	−0.04	.84	1.1%
AC	−0.9	−0.33	.09	1.0%	−1.7	−0.22	.26	1.3%
cAB	−1.0	−0.29	.14	1.0%	−3.0	−0.36	.07	1.1%
VCtAB	−2.7	−0.59	.00	1.9%	−4.0	−0.33	.09	2.0%
ThCtAB.aMe	−2.6	−0.58	.01	1.9%	−3.6	−0.31	.12	1.7%
ThCtAB.bMe	−2.8	−0.62	.00	1.9%	−3.3	−0.29	.13	1.9%
ThCtAB.cMe	−2.5	−0.56	.01	2.0%	−3.9	−0.33	.10	1.7%
ThCcAB.aMe	−1.4	−0.43	.03	1.9%	−1.5	−0.18	.34	1.7%
ThCcAB.bMe	−1.5	−0.48	.02	1.9%	−1.2	−0.15	.43	1.9%
ThCcAB.cMe	−1.3	−0.42	.04	2.0%	−2.0	−0.23	.23	1.7%
ThCtAB.aMax	−1.4	−0.27	.17	4.4%	0.0	0.00	.99	2.8%
ThCtAB.bMax	−1.7	−0.30	.12	4.2%	0.7	0.10	.61	3.3%
ThCtAB.cMax	−1.1	−0.18	.35	5.3%	−0.5	−0.09	.65	3.2%
ThCtAB.aMav	−1.4	−0.31	.11	3.8%	−0.3	−0.05	.79	2.5%
ThCtAB.bMav	−1.7	−0.35	.07	3.5%	0.0	0.00	.98	2.8%
ThCtAB.cMav	−1.3	−0.25	.19	4.5%	−0.5	−0.09	.65	2.8%

MC%: mean change in %, SRM: standardized response mean (= mean change/SD of change), *P*: level of significance of change using a paired *t*-test without adjustment for multiple comparisons; RMSCV%: root mean square coefficient of variation of test-retest acquisitions at baseline, with repositioning in between scans. For other abbreviations, please see Table 1. Note that values are given for the “long” femoral region of interest, that is, a 60% distance between the trochlear notch and the posterior end of both femoral condyles.
